# Two Pilot Studies of the Effect of Bicycling on Balance and Leg Strength among Older Adults

**DOI:** 10.1155/2013/686412

**Published:** 2013-04-17

**Authors:** Chris Rissel, Erin Passmore, Chloe Mason, Dafna Merom

**Affiliations:** ^1^Prevention Research Collaboration, School of Public Health, University of Sydney, Sydney, NSW 2050, Australia; ^2^University of Sydney, Council on The Ageing (NSW), Pitt Street, Sydney, NSW 2000, Australia; ^3^New South Wales Ministry of Health, Miller Street, North Sydney, NSW 2060, Australia; ^4^University of Western Sydney, Campbelltown Campus, Campbelltown, NSW 2560, Australia

## Abstract

*Objectives*. Study 1 examines whether age-related declines in balance are moderated by bicycling. Study 2 tests whether regular cycling can increase leg strength and improve balance. *Methods*. *Study 1*: a cross-sectional survey of 43 adults aged 44–79 was conducted. Leg strength was measured, and Balance was measured using the choice stepping reaction time (CSRT) test (decision time and response time), leg strength and timed single leg standing. *Study 2*: 18 older adults aged 49–72 were recruited into a 12-week cycling program. The same pre- and postmeasures as used in Study 1 were collected. *Results*. *Study 1*: participants who had cycled in the last month performed significantly better on measures of decision time and response time. *Study 2*: cycling at least one hour a week was associated with significant improvements in balance (decision time and response time) and timed single leg standing. *Conclusions*. Cycling by healthy older adults appears promising for improving risk factors for falls.

## 1. Introduction

Falls in older people represent a significant health burden. One-third of adults aged over 65 years fall at least once each year [1]. Falls can result in injuries (e.g., fractures), loss of confidence, and restriction of physical activity [2]. Physiological risk factors have been found to be associated with greater risk of falling, include gait instability, leg weakness, and poor balance [3].

The deterioration of balance with age has been well documented [4], and physical activities that increase balance and strength are recommended for falls prevention for older adults [5]. A recent meta-analysis found that the most effective physical interventions for falls prevention involve a challenge to balance [6].

The majority of physical activity interventions for falls prevention are structured, supervised group training programs. However, participation in these programs is variable, and more than half of older Australians undertake their physical activity in unstructured forms [7]. Studies from the USA and Europe suggest that many older adults prefer to exercise alone rather than in organised group classes [8], and that the cost, time, and difficulty of travel to group programs can be barriers to participation [9]. Identifying activities that are enjoyable and accessible for older people that have a benefit in contributing to falls prevention is the challenge, and there is currently limited evidence about the effect of recreational leisure time or daily physical activity on falls risk factors [10]. To date, Tai Chi is the only single physical activity that has been tested and shown to prevent falls through its positive effect on strength and dynamic balance [11, 12].

Riding a bicycle is an example of an increasingly popular physical activity for older adults; in Australia, increasing numbers of older adults cycled for recreation and transport in the past decade [7, 13]. Cycling is a healthy form of physical activity [14] and a non-weight bearing activity that is less stressful on the body than jogging or other running sports. A recent systematic review found a consistent positive relationship between cycling and cardiorespiratory fitness, functional abilities, and disease risk factor profiles [15]. Several longitudinal epidemiological studies have shown significant risk reduction for all-cause and cancer mortality and for cardiovascular disease, colon and breast cancer, and obesity morbidity in middle-aged and elderly men and women [15]. Cycling may also have other benefits for older people, such as providing opportunities for recreation and socialising, and as an affordable form of transport.

There are risks associated with cycling, especially traumatic injuries. However, a recent analysis has compared the risks and benefits of shifting from a car to bicycle commuting in urban settings and estimated that the life expectancy gained due to increased physical activity (3–14 months gained) was far greater than the life expectancy lost due to increased air pollution (0.8–40 days lost) and traffic injury (5–9 days lost) [16].

It is not known if cycling prevents falls. However, there is some evidence that cycling is associated with increased leg strength and balance, important risk factors for falling. Indoor stationary cycling has been found to increase leg strength, muscle endurance, balance, and functional abilities in healthy middle-aged and older adults [17–19]. Although the impact of nonstationary cycling has not been tested in older adults, we expect that nonstationary cycling would also increase leg strength and balance, and it may have additional benefits such as increased social activity.

This paper reports on two pilot studies exploring the relationship between cycling participation and leg strength and balance in healthy older adults. The first pilot study examines the cross-sectional association of recent cycling with leg strength and balance, and the second pilot study examines whether an older adult noncyclist riding a bicycle will increase leg strength and improve balance.

## 2. Study 1 Methods

The design of this pilot study is a cross-sectional study. Participants were recruited by invitations circulated through existing cycling and social networks. Eligible participants were healthy adults aged over 40 years and gave informed consent. Both Study 1 and Study 2 were approved by the University of Sydney Ethics Review Committee.

### 2.1. Measures

#### 2.1.1. Questionnaire

A short questionnaire was administered orally by one of the investigators. Participants were asked when they last rode a bicycle, and how often they usually cycled. Recent cycling (in the past month) was considered the key independent variable. Participants were also asked to describe their regular physical activities. The investigators then assigned a crude rating of physical activity on a scale from 1 to 10 (1 = no physical activity, 10 = frequent, intense physical activity). Demographic details were also collected.

#### 2.1.2. Physical Measures

*Seated Leg Strength.* The strength of three leg muscle groups (knee flexors and extensors and ankle dorsiflexors) was measured, while participants were seated. An electronic force gauge (Checkline Model Series 4) was attached to the participant's leg and secured to the back of the chair. Participants were then asked to straighten their leg out from the chair as far as possible. There were three trials on each leg. For each trial, the greatest force was recorded (to a maximum of 50 kg). Quadriceps strength using a spring gauge to measure knee extension has been shown to have a good intra-rater reliability (ICC = 0.76) [20].

*Dynamic Balance.* Dynamic balance was measured using the choice step reaction time (CSRT) test.

The CSRT test uses a plastic mat marked with two central footprints and four arrows (one in front of each foot and one to the side of each foot). The participant stands facing a computer screen displaying a diagram of the mat. On the screen, arrows are illuminated in random order, and participants step on the corresponding arrow on the mat as quickly as possible. Two measures were collected: decision time (time between arrow being illuminated on screen and participant raising foot from centre footprint) and response time (time between raising the foot from centre footprint to placing foot on correct arrow). The Choice Step Reaction Time test is associated with a range of sensorimotor and balance measures important in preventing falls, and is also effective at differentiating fallers from nonfallers [21]. 

*Standing Balance.* Standing balance was measured using the timed single leg standing test [22]. Participants were asked to stand on one leg with eyes open for as long as possible up to a maximum of 5 minutes. There was one trial on each leg.

*Statistical Analysis.* Participants' best trials for standing balance and leg strength were included in analyses. For the Choice Step Reaction Time test, mean decision time and response time were calculated. Regression models were created with four separate outcome measures: standing balance, leg strength, dynamic balance decision time, and dynamic balance response time. The main variable of interest was cycling activity in previous month (yes/no). Three other potentially confounding variables were also included in the regression models: age, sex, physical activity rating (0–10). The data were analysed using SPSS Version 19.

## 3. Study 1 Results

There were 43 participants; 67% were female. Participants ranged in age from 44 to 79 years (mean = 57.2, standard deviation = 7.9). Twelve participants (28%) rated their current health status as excellent, 20 (47%) as very good, 9 (21%) as good, and 2 (5%) as poor.

Current participants physical activity ratings ranged from 1 to 10 (mean = 5.4, standard deviation = 2.5). Forty percent of participants (*n* = 17) had cycled within the past month, a further 15% within the last year, and the rest longer than a year.

As expected, performance on two of the four measures of strength and balance significantly declined with age. These were leg strength (regression coefficient = −0.592, *P* = 0.008) and standing balance (regression coefficient = −6.58, *P* = 0.001). Decision times increased marginally (regression coefficient = 0.005, *P* = 0.009), and response time was not associated with age (regression coefficient = 0.007, *P* = 0.187). In contrast, performance on all four measures improved significantly with increasing physical activity. After adjusting for age, sex, and physical activity rating, participants who had cycled in the last month (“cyclists”) performed significantly better on measures of decision time (*P* = 0.008) and response time (*P* = 0.050) compared to those who had not cycled in the last month (“noncyclists”) (see Table 1). There was a borderline significant difference in standing balance between cyclists and noncyclists (*P* = 0.063).

Cycling was not a statistically significant independent predictor of timed one leg standing balance; however, the 62 seconds difference in standing balance between cyclists and noncyclists is potentially clinically significant. Cycling was negatively associated with leg strength, with noncyclist's average leg strength significantly greater than cyclists. 

## 4. Study 2 Methods

The design of study two was a pre-post program evaluation. We sought to recruit adults aged from 50 to 75 years who were willing to cycle for two or more hours per week over a 12-week period. This amount of activity has been assessed as the minimum amount necessary for an intervention effect for preventing falls [5]. Eligible participants were healthy adults who were able to cycle, had cycled previously but were currently not cycling (average of less than 30 minutes a week), and gave informed consent.

### 4.1. Program Components

The program was initially situated in a discrete geographic area of Sydney (Canada Bay) and commenced with a cycling skills course to develop participants cycling ability and confidence. The course was delivered by accredited trainers “Bikewise,” and funded by City of Sydney Council. The course duration was 4.5 hours, and it covered the basic bicycle control skills (starting, braking, and turning), route planning, and practiced on-road group and individual riding skills. Participants were then encouraged to cycle for at least two hours a week over 12 weeks.

Participants were also supported by mentors in their local area over the 12 weeks of their participation and received a resource pack with information about cycling. The research team made brief informal phone calls to participants at one and six weeks after the cycle skills course to maintain participant engagement with the project and identify any additional support required. The project timeline is shown in Table 2.

Measures of leg strength and balance were collected, as described for Pilot Study 1. These were taken before the cycling skills course and at 12 weeks. Participants were also asked to keep a diary recording their cycling. At the end of the project, participants were asked a series of open-ended questions about their experiences of cycling, with the interviews being recoded and analysed qualitatively for major themes.

### 4.2. Statistical Analysis

Quantitative outcome measures were single leg standing balance time (seconds), leg strength (kilograms), and decision time and response time on the Choice Step Reaction Time test (seconds). Analysis was a pre-post comparison of leg strength and balance using paired sample *t*-tests. Participants' best trial for standing balance and leg strength was included in analysis. For the Choice Step Reaction Time test, mean decision time and response time were calculated. The data were analysed using SPSS Version 19.

## 5. Study 2 Results

There were 18 participants, of whom 13 were female. Participants ranged in age from 49 to 72 years, with an average age of 61. At baseline, six participants rated their current health status as excellent, seven as very good, and five as good. When asked at baseline about the time since they last cycled, eleven participants had not cycled in the last year, three had cycled in the last year, three in the last month, and one in the last week, with recent cyclists riding less than 30 minutes a week.

*(1) Cycling Participation.* Two participants withdrew from the study (for personal reasons), one was unable to participate due to injury, a fourth could not be contacted for timely followup, and two more participants had only done a very small amount of cycling (less than 10 minutes a week). All withdrawals were female. This left 12 participants (66%) who had on average cycled an hour or more a week for the past 12 weeks (including six who cycled for more than two hours a week), and for whom baseline and follow-up information was available. Five of these remaining participants were male, and seven were female. The average age (SD) was 62.2 (4.6) years and ranged from 53 to 68 years.

*(2) Physical Measures.* Pre- and postintervention results for the twelve participants who cycled for at least an hour per week are presented in Table 3. Overall, participants significantly improved their performance on measures of one leg standing balance (*P* = 0.028), and response time (*P* = 0.031), and borderline significance for decision time (*P* = 0.058). Leg strength increased over the 12 weeks, but not significantly.

*(3) Qualitative Feedback.* From participant feedback during the follow-up data collection, the program appears to have been successful in supporting most participants to start cycling regularly. Several participants described the program as a “life-changing” experience that made it possible to achieve their goal of cycling. Participants described health benefits including increased fitness, increased leg strength, weight loss, and improved performance in other physical activities such as walking and running. Cycling was also considered beneficial for participants' mental health, as a form of relaxation. Participants identified social benefits of cycling, reporting that group rides had expanded their social network, or that cycling was an activity they could enjoy with their partner and friends.

Almost all participants said the skills course was essential in developing their cycling skills and confidence, especially for riding in groups and on roads. Participants also found it helpful to have “mentoring” from another local cyclist who was able to provide tips, support, and encouragement.

## 6. Discussion

We found that older adults who had cycled recently and those who cycled for over an hour a week performed better than nonriders or infrequent cyclists on measures of static and dynamic balance, important predictors of falls risk. After adjusting for age, sex, and physical activity, cycling was a significant predictor of decision time and response time on a dynamic balance task.

Although cycling was not a statistically significant independent predictor of standing balance time, this may reflect the limited power of the study due to the small sample size. Cyclists balanced on one leg for 62 seconds longer on average than noncyclists, a large and potentially clinically significant difference. The clinical significance of this finding is difficult to judge as methods for administration of the one-legged standing test vary greatly between studies; however, many studies have found that decreased one-legged standing time is associated with increased falls risk and declines in activities of daily living [23].

Our finding of no association between cycling and leg strength in Study 1 was unexpected, although may be due to the way leg strength was measured. Gains in strength are in part specific to the type of muscle action used in training [24]. Cycling involves using several leg muscle groups at relatively low force over a long period of time, whereas the leg strength test involved exerting maximum force for a short period using mainly quadriceps muscles. Despite a small increase in leg strength as a consequence of regular cycling, other studies also have found that stationary cycling does not improve seated leg strength in healthy middle-aged women [19] or stroke patients [25]. In addition, although leg weakness is a risk factor for falls, there is no evidence that leg strength training reduces falls risk [6]. It is possible that once a person has sufficient leg strength to avoid falling, further strength training has no additional benefit [6].

In addition to the measured balance improvements, participants also reported a range of other benefits from cycling, including improved health and wellbeing, increased confidence, and expanded social opportunities. Most project participants were reasonably physically active before they started the program, and the benefits of cycling may be even greater for people with lower baseline levels of physical activity.

Overall, the results suggest that cycling is a potential falls prevention strategy for healthy older adults who are able to ride a bicycle. Previous research indicates that exercise can protect against age-related decline in strength and balance and reduce the risk of falls [5]. However, this is one of the first studies to specifically examine the relationship between outdoor bicycling and leg strength and balance in healthy older adults and to have demonstrated improvements related to regular cycling. These preliminary results suggest that cycling has an additional impact on balance, over and above what is gained through other physical activity. Thus, cycling may further reduce falls risk even among adults who are already physically active.

The main limitations of these pilot studies are the small sample sizes in both studies, the lack of a comparison group for Study 2, lack of sensitivity to cycling of the leg strength measure, and the use of a nonvalidated measure of physical activity. However, with the effect sizes shown, sample size calculations can be made for future research to replicate these results in a larger sample. Further research is required to verify the findings of this pilot program. Larger-scale trials over a longer time period are required to replicate these findings and examine whether the observed improvements in balance translate to reduced rates of falls.

Given the many benefits of cycling, we recommend that cycling programs for older people become more widely available. Our program provides a successful model for supporting older adults to cycle that could be easily replicated elsewhere, and it was delivered at low cost by making use of existing resources (i.e., local Bicycle User Group and a free cycling skills course) plus a coordinating role. Key elements of our program that participants identified as helpful were the skills course and mentoring, and we suggest future initiatives to promote cycling for older adults incorporate these components as a way of developing cycling skills and confidence in a supportive peer environment. Dedicated cycling courses for older adults or “rusty riders” could facilitate greater participation. Rider support through mentors, that is, being able to ride with someone else who could develop their safety and riding skills and show them safe local routes, was important for older adults. Support for local Bicycle User Groups (who organise social and community rides and participate in advocacy) is a practical way to provide this mentoring.

In addition, it is important to continue to expand cycling infrastructure such as dedicated off-road cycle paths, separated cycleways, and connectivity to other forms of public transport. This would allow new riders of all ages to access off-road cycle areas, where they feel safe to develop their skills and potentially transition to on-road riding.

## Key Points

- Adults who had cycled in the last month performed significantly better on balance measures of decision time and response time.
- Cycling at least one hour a week was associated with significant improvements in balance (decision and response times) and timed single leg standing.
- Cycling by healthy older adults appears promising for improving risk factors for falls.

## Figures and Tables

**Table 1:** Mean leg strength and balance outcome measures of participants who had cycled in the past month (cyclists) and those who had not (noncyclists) (*n* = 43).

Measure	Noncyclist (n = 26)	Cyclist (n = 17)	*P* ^†^
	mean (SD)	mean (SD)	
Leg strength (kg)	37.4 (11.8)	31.9 (11.0)	0.012
Standing balance (sec)	156.3 (113.7)	218.3 (89.1)	0.063
Dynamic balance decision time (sec)	0.67 (0.12)	0.58 (0.05)	0.008
Dynamic balance response time (sec)	0.97 (0.31)	0.79 (0.09)	0.050

^†^Adjusting for age, sex, and other physical activity.

**Table 2:** Timeline for Older People Cycling Project.

Dates	Activities
Oct-Nov 2011	Promotion of project in Canada Bay Council area through Drummoyne Community Centre, City of Canada Bay Council, Canada Bay Bicycle User Group, and inviting people to volunteer as mentors and participants
Dec 2011–March 2012	Enrolment of mentors and participants
Jan–April 2012	Baseline interview, questionnaire, and physical measures. Participation in cycling skills course
12 weeks	Participants ride two hours a week over at least two days, recording rides in diary
April–June 2012	Follow-up interview and physical measures

**Table 3:** Pre-and postintervention leg strength and balance for participants cycling one hour or more per week over 12 weeks (*n* = 12).

Measure	Preprogram	Postprogram	*P*
	mean (SD)	mean (SD)	
Leg strength (kg)	31.4 (11.6)	36.2 (10.3)	0.141
Standing balance (sec)	145.7 (90.7)	175.2 (98.5)	0.028
Dynamic balance decision time (sec)	0.62 (0.04)	0.59 (0.04)	0.058
Dynamic balance response time (sec)	0.86 (0.05)	0.81 (0.05)	0.031

## References

[1] Lord SR, Ward JA, Williams P, Anstey KJ (1993). An epidemiological study of falls in older community-dwelling women: the Randwick falls and fractures study. *Australian Journal of Public Health*.

[2] Vellas BJ, Wayne SJ, Romero LJ, Baumgartner RN, Garry PJ (1997). Fear of falling and restriction of mobility in elderly fallers. *Age and Ageing*.

[3] Rubenstein LZ (2006). Falls in older people: epidemiology, risk factors and strategies for prevention. *Age and Ageing*.

[4] Camicioli R, Panzer VP, Kaye J (1997). Balance in the healthy elderly: posturography and clinical assessment. *Archives of Neurology*.

[5] Sherrington C, Tiedemann A, Fairhall N, Close J, Lord SR (2011). Exercise to prevent falls in older adults: an updated meta-analysis and best practice recommendations. *NSW Public Health Bulletin*.

[6] Sherrington C, Whitney JC, Lord SR, Herbert RD, Cumming RG, Close JCT (2008). Effective exercise for the prevention of falls: a systematic review and meta-analysis. *Journal of the American Geriatrics Society*.

[7] Merom D, Cosgrove C, Venugopal K, Bauman A (2012). How diverse was the leisure time physical activity of older Australians over the past decade?. *Journal of Science and Medicine in Sport*.

[8] Wilcox S, King AC, Brassington GS, Ahn DK (1999). Physical activity preferences of middle-aged and older adults: a community analysis. *Journal of Aging and Physical Activity*.

[9] Yardley L, Bishop FL, Beyer N (2006). Older people’s views of falls-prevention interventions in six European countries. *Gerontologist*.

[10] Nelson ME, Rejeski WJ, Blair SN (2007). Physical activity and public health in older adults: recommendation from the American College of Sports Medicine and the American Heart Association. *Medicine and Science in Sports and Exercise*.

[11] Voukelatos A, Cumming RG, Lord SR, Rissel C (2007). A randomized, controlled trial of tai chi for the prevention of falls: the central sydney tai chi trial. *Journal of the American Geriatrics Society*.

[12] Logghe IHJ, Verhagen AP, Rademaker ACHJ, Bierma-Zeinstra SMA, Faber MJ, Koes BW (2010). The effects of Tai Chi on fall prevention, fear of falling and balance in older people: a meta-analysis. *Preventive Medicine*.

[13] Merom D, van der Ploeg HP, Corpuz G, Bauman AE (2010). Public health perspectives on household travel surveys: active travel between 1997 and 2007. *American Journal of Preventive Medicine*.

[14] Bauman AE, Rissel C (2009). Cycling and health: an opportunity for positive change?. *Medical Journal of Australia*.

[15] Oja P, Titze S, Bauman A (2011). Health benefits of cycling: a systematic review. *Scandinavian Journal of Medicine and Science in Sports*.

[16] de Hartog JJ, Boogaard H, Nijland H, Hoek G (2010). Do the health benefits of cycling outweigh the risks?. *Environmental Health Perspectives*.

[17] Bouillon LE, Sklenka DK, Ver ACD (2009). Comparison of training between 2 cycle ergometers on dynamic balance for middle-aged women. *Journal of Sport Rehabilitation*.

[18] Vilarinho R, de Souza WY, Rodrigues TC, Ahlin JV, Guedes DP, Barbosa FM (2009). Effects of indoor cycling in body composition, muscular endurance, flexibility, balance and daily activities in physically active elders. *Fitness & Performance Journal*.

[19] Sillanpää E, Häkkinen A, Laaksonen DE, Karavirta L, Kraemer WJ, Häkkinen K (2010). Serum basal hormone concentrations, nutrition and physical fitness during strength and/or endurance training in 39–64-year-old women. *International Journal of Sports Medicine*.

[20] Sherrington C, Lord SR (2005). Reliability of simple portable tests of physical performance in older people after hip fracture. *Clinical Rehabilitation*.

[21] Lord SR, Fitzpatrick RC (2001). Choice stepping reaction time: a composite measure of falls risk in older people. *Journals of Gerontology A*.

[22] Rossiter-Fornoff JE, Wolf SL, Wolfson LI (1995). A cross-sectional validation study of the FICSIT common data base static balance measures. *Journals of Gerontology A*.

[23] Michikawa T, Nishiwaki Y, Takebayashi T, Toyama Y (2009). One-leg standing test for elderly populations. *Journal of Orthopaedic Science*.

[24] Fleck SJ, Kraemer WJ (2004). *Designing Resistance Training Programs*.

[25] Janssen TW, Beltman JM, Elich P (2008). Effects of electric stimulation-assisted cycling training in people with chronic stroke. *Archives of Physical Medicine and Rehabilitation*.

